# PReS-FINAL-1006: Autologous bone marrow transplantation in autoimmune, experimental arthritis restores immune homeostasis by renewal of the natural tregs compartment

**DOI:** 10.1186/1546-0096-11-S2-P4

**Published:** 2013-12-05

**Authors:** T van den Broek, E Delemarre, J Meerding, E Wehrens, F Broere, N Wulffraat, M Boes, B Prakken, F van Wijk

**Affiliations:** 1 Pediatric Immunology, Lab Translational Immunology (LTI), Wilhelmina Children's Hospital, University Medical Center Utrecht, The Netherlands; 2 Institute of Infectious Diseases and Immunology, Utrecht University, Utrecht, Utrecht, The Netherlands

## Introduction

Autologous bone marrow transplantation (aBMT) is a last resort treatment for patients with refractory juvenile idiopathic arthritis (JIA). It can induce disease remission for 80 months after transplantation. Unfortunately, relapses also occur. The underlying working mechanisms remain largely unknown.

## Objectives

We investigated the potential role of regulatory T cells (Treg) during immune reconstitution and re-establishment of immune tolerance following aBMT.

## Methods

Arthritis was induced by two intraperitoneal injections of proteoglycan (PG) in synthetic adjuvant dimethyl dioctadecyl ammonium bromide (DDA), two and five weeks before stem cell transplantation. The onset and severity of arthritis were assessed three times a week in a blinded fashion by a visual scoring system. Two weeks after the second PG/DDA injection, a lethal dose of 7.5 Gy total body irradiation was given to arthritic mice. Recipient mice were injected with 2 × 10^6 ^bone marrow (BM) cells. GFP+Treg cells were sorted by flow cytometry as TCRb+ CD4+ CD25+ GFP+ T cells. Before infusion, Treg suspensions were added to the bone marrow graft in different amounts.

## Results

In the PGIA mouse model, lethal irradiation followed by aBMT, reduced arthritis scores and restored the immune balance between pro-inflammatory effector T cells and Treg. Directly following aBMT the majority of Treg present, consisted of Treg that survived the conditioning. Here after, the infused stem cell-derived Treg started dominating the Treg pool and these "new" thymus-derived Treg showed more suppressive capacity than the remaining host Treg. A therapeutic approach was initiated by infusing extra Foxp3^GFP^Treg together with the stem cell graft. The infused Treg expanded vigorously in the first month after aBMT, followed by a decrease in numbers in the second month. No extra clinical improvement was found in the Treg-infused groups, the highest Treg dosed group even showed an increase in disease relapse. Both Treg treated groups showed delayed induction of ' new' stem cell derived Treg.

## Conclusion

These data indicate that restoration of the immune balance following aBMT depends on renewal of the natural Treg pool derived from the injected stem cells. For now, infusion of extra Treg during aBMT is not recommended as this may delay T cell reconstitution and development of long-term tolerance.

## Disclosure of interest

None declared.

